# Concurrent Validity, Test-Retest Reliability, and Sensitivity to Change of a Single Body-Fixed Sensor for Gait Analysis during Rollator-Assisted Walking in Acute Geriatric Patients

**DOI:** 10.3390/s20174866

**Published:** 2020-08-28

**Authors:** Christian Werner, Patrick Heldmann, Saskia Hummel, Laura Bauknecht, Jürgen M. Bauer, Klaus Hauer

**Affiliations:** 1 Center for Geriatric Medicine, Heidelberg University, 69117 Heidelberg, Germany; juergen.bauer@bethanien-heidelberg.de; 2 AGAPLESION Bethanien Hospital Heidelberg, Geriatric Center at the Heidelberg University, 69126 Heidelberg, Germany; khauer@bethanien-heidelberg.de; 3 Network Aging Research (NAR), Heidelberg University, 69117 Heidelberg, Germany; heldmann@nar.uni-heidelberg.de; 4 Medical Faculty Heidelberg, Heidelberg University, 69117 Heidelberg, Germany; saskiahum@gmail.com (S.H.); bauknecht@stud.uni-heidelberg.de (L.B.)

**Keywords:** gait analysis, inertial measurement unit, wearable sensors, wheeled walker, spatio-temporal parameters, validation, geriatrics, elderly

## Abstract

Body-fixed sensor (BFS) technology offers portable, low-cost and easy-to-use alternatives to laboratory-bound equipment for analyzing an individual’s gait. Psychometric properties of single BFS systems for gait analysis in older adults who require a rollator for walking are, however, unknown. The study’s aim was to evaluate the concurrent validity, test-retest-reliability, and sensitivity to change of a BFS (DynaPort MoveTest; McRoberts B.V., The Hague, The Netherlands) for measuring gait parameters during rollator-assisted walking. Fifty-eight acutely hospitalized older patients equipped with the BFS at the lower back completed a 10 m walkway using a rollator. Concurrent validity was assessed against the Mobility Lab (APDM Inc.; Portland, OR, USA), test-retest reliability over two trials within a 15 min period, and sensitivity to change in patients with improved, stable and worsened 4 m usual gait speed over hospital stay. Bland–Altman plots and intraclass correlation coefficients (ICC) for gait speed, cadence, step length, step time, and walk ratio indicate good to excellent agreement between the BFS and the Mobility Lab (ICC_2,1_ = 0.87–0.99) and the repeated trials (ICC_2,1_ = 0.83–0.92). Moderate to large standardized response means were observed in improved (gait speed, cadence, step length, walk ratio: 0.62–0.99) and worsened patients (gait speed, cadence, step time: −0.52 to −0.85), while those in stable patients were trivial to small (all gait parameters: −0.04–0.40). The BFS appears to be a valid, reliable and sensitive instrument for measuring spatio-temporal gait parameters during rollator-assisted walking in geriatric patients.

## 1. Introduction

Gait disorders are prevalent in older adults [1,2], and have been associated with a higher risk of adverse outcomes such as falls, disability, institutionalization, and mortality [2,3,4,5]. A particularly high prevalence of gait disorders can be expected in hospitalized older patients [6,7], who are typically characterized by multimorbidity, geriatric conditions (e.g., cognitive impairment, polypharmacy, malnutrition) and/or frailty [8,9,10]. Improving a patient’s ability to walk safely and independently is, therefore, a relevant goal of geriatric rehabilitation [11]. Consequently, quantitative gait analysis in geriatric settings has become increasingly important for identifying a patient’s gait disorders, guiding clinical decisions, customizing treatment, and monitoring the effectiveness of interventions [12,13]. Optical motion-capture systems, force plates, or instrumented walkways are established gold standards for quantitative gait analysis [12,14,15]; however, these systems are expensive, require time-consuming set-ups and manual post processing, and/or are constrained to laboratory environments, limiting their accessibility and feasibility in both clinical practice and research [16,17,18]. Recent advances in wearable sensor technology provide clinicians and researchers with more affordable, easy-to-use and highly portable alternatives for measuring an individual’s spatio-temporal gait parameters during walking [19,20,21,22]. Such wearable or body-fixed sensor (BFS) systems for quantitative gait analysis most frequently incorporate miniaturized inertial measurement units (IMUs), which consist of tri-axial accelerometers, gyroscopes and, in some cases, magnetometers [23,24]. Some of these BFS systems are based on multiple IMUs (e.g., APDM Mobility Lab, BioSensics LEGSys) attached to various parts of an individual’s body (e.g., feet, knees, thighs or waist), while others only include a single IMU to collect gait data (e.g., DynaPort MoveTest [MT]). In recent years, such BFS systems have become a common alternative to the expensive and strictly lab-based methods for comprehensive gait analysis in laboratory, clinical, and daily living environments [18,19,21,25,26]. An increasing number of studies also indicate that BFS systems are valid and reliable for quantifying spatio-temporal gait parameters in healthy older adults (for review see [27]) and different patient populations (e.g., individuals with Parkinson’s disease and other neurological disorders [28,29,30,31,32], mild cognitive impairment [29], lower-limb prosthesis [33], or hip osteoarthritis and total hip replacement [34]). Although BFS systems have already been used for gait analysis in older adults during walking with a rollator (for review see, [35]), there is limited evidence on their psychometric properties for quantifying spatio-temporal gait parameters (e.g., gait speed, cadence, step time, or step length) under this walking condition. Older adults with walking aids were most frequently rather excluded from validation studies of BFS systems for gait analysis [29,33,36,37,38,39,40,41]. We could identify only one study that examined the concurrent validity of a BFS-based gait analysis system (eGaIT) consisting of two IMUs (Shimmer 2R) attached to the shoes against an instrumented walkway (GAITRite) during rollator-assisted walking in geriatric patients [42]. However, this study did not investigate further psychometric properties such as test-retest reliability or sensitivity to change, which are essential for differentiating between real change and random measurement error and for determining the ability of a measurement to detect changes over time or to monitor the effectiveness of interventions. To the best of our knowledge, there is currently no comprehensive clinical evaluation on the validity, reliability, and sensitivity to change of a single BFS system for gait analysis during rollator-assisted walking in older adults.

Walking aids such as rollators are frequently prescribed in geriatric rehabilitation to compensate for gait disorders and postural instability, to enhance patient safety and decrease fall risk, to reduce the dependency on caregivers, and to support independent ambulation and mobility [42,43,44,45]. Geriatric acute patients with advanced gait impairments are often unable to walk without a rollator [46,47], even over short distances, so that a quantitative gait analysis in such patients can only be conducted during rollator-assisted walking. Consequently, there is also a need for valid, reliable, and sensitive BFS system for gait analysis in rollator users to evaluate treatment or intervention effects on their spatio-temporal gait parameters and to avoid invalid or missing data in clinical practice and research.

In summary, the aim of this study was to evaluate the concurrent validity, test-retest-reliability, and sensitivity to change of a single BFS system (DynaPort MT; McRoberts B.V., The Hague, The Netherlands) for measuring spatio-temporal gait parameters during rollator-assisted walking in acutely hospitalized geriatric patients.

## 2. Materials and Methods

### 2.1. Study Design

The present validation study was a secondary analysis of data collected as part of a prospective, longitudinal cohort study on physical activity and mobility in acutely hospitalized older patients (“Physical Activity in Geriatric patients during Early Rehabilitation”, PAGER). The PAGER study was conducted between January 2019 and August 2019, in accordance with the Declaration of Helsinki. Written informed consent was obtained from all participants (or legal representatives) prior to study inclusion. The Ethics Committee of the Medical Faculty of Heidelberg approved the study protocol (S-709/2018). The PAGER study was registered at the German Clinical Trials Register (DRKS00016028).

### 2.2. Study Population and Setting

Participants were consecutively recruited from the acute medical inpatient units of a German geriatric hospital (AGAPLESION Bethanien Hospital, Geriatric Center at the Heidelberg University, Germany). Acute geriatric patients receiving complex early geriatric rehabilitation treatment according to the German hospital payment system (German Diagnosis-Related Groups (G-DRGs): Classification of Operations and Procedures (OPS) code 8-550.1) were included in the PAGER study. The early geriatric rehabilitation treatment (OPS code 8-550.1) is a multidisciplinary inpatient rehabilitation program with at least 14 treatment days and 20 therapy sessions provided by a geriatric team under the direction of a geriatric physician. Therapy sessions last on average 30 min and address at least two of the following four therapeutic domains, depending on the patients’ individual needs: physiotherapy, occupational therapy, speech and language therapy, and psychotherapy [48]). Further inclusion criteria were as follows: 65 years or older, Mini-Mental State Examination (MMSE, [49]) score ≥ 10, ability to walk at least 4 m with or without a walking aid, no severe somatic or psychiatric disorders, no delirium, no isolation, adequate German language skills, and written informed consent within 72 h after hospital admission.

The present validation study only included PAGER participants with gait and balance disorders that used the specific walking aid of a rollator during hospital stay, as it has been shown that various walking aids can have different effects on gait parameters in older adults [35], which thus could have been affected in the study results. The sample size for this secondary data analysis was estimated to be at least 57 participants, based on a prior analysis for the concurrent validity of the BFS with an expected intraclass correlation coefficient (ICC) of 0.9 for two measurements (*k* = 2), a 95% confidence interval (CI) width of 0.1, and an alpha level (α) of 0.05 [50,51].

### 2.3. Descriptive Measures

Sociodemographic and clinical characteristics of participants were assessed as early as possible after hospital admission (T1). Age, gender, and comorbidity (number of diagnoses and medications) were documented from patient charts. Trained interviewers assessed falls in the previous year, cognitive status (MMSE), depressive symptoms (Geriatric Depression Scale, 15-item version, GDS) [52], fear of falling (Falls Efficacy Scale-International, 7-item version, FES-I) [53], functional status in activities of daily living (Barthel Index) [54]. Physical capacity measures administered by a trained physical therapist included the Short Physical Performance Battery (SPPB) [55], 4 m gait speed test (as part of the SPPB), the De Morton Mobility Index (DEMMI) [56], and handgrip strength (Jamar hydraulic hand dynamometer) [57].

### 2.4. Gait Measurements

Gait analyses were performed at T1 and at the end of complex early geriatric rehabilitation treatment as close as possible to hospital discharge (T2). All gait analyses took place in the laboratory environment within the AGAPLESION Bethanien Hospital and were administered by a physical therapist with 16 years working experience, supported by a medical student to ensure the safety of the participants.

#### 2.4.1. Test Procedure

Participants were asked to walk once along a 10 m walkway at self-selected comfortable walking speed using a standard 4-wheeled walker (rollator) with two front steering wheels (360° rotatable) and two fixed rear wheels (Allround Rollator; Samhall Rehab AB, Malmo, Sweden). Each walk was initiated and terminated 2 m before and after the walkway to minimize effects of acceleration and deceleration (total length = 14 m). The handle height of the rollator was adjusted to each participant’s individual wrist height while standing upright with arms resting vertically on the side of the body. No practice trials were performed on the walkway prior to data collection.

During the test session at T1, a retest was performed following a 15 min rest period after the first test. To simulate application of the BFS during routine clinical assessment, the elastic strap with the BFS was detached and reattached between the pre- and retest.

#### 2.4.2. Instrumentation and Data Collection

**Figure 1:**
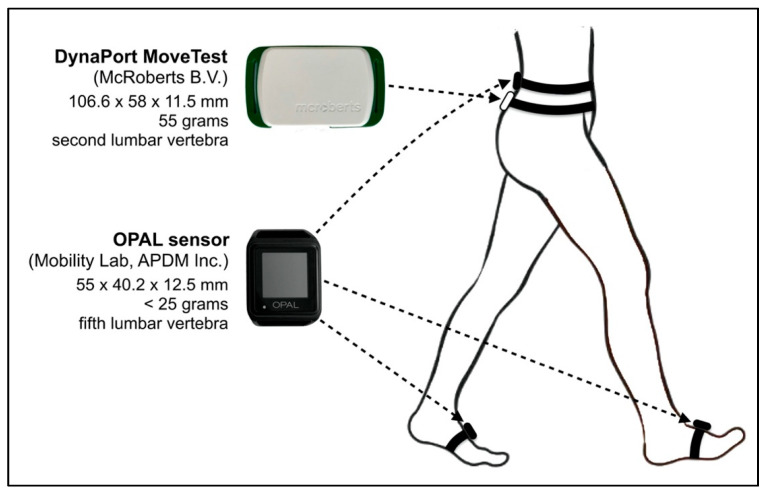
Placement of the DynaPort MoveTest and the OPAL sensors of the Mobility Lab.

The single BFS (DynaPort MT; McRoberts B.V., The Hague, The Netherlands) attached to an elastic strap was positioned on the participants’ lower back over the second lumbar vertebra (=approximation of the body’s center of mass in the standing position; Figure 1). The small and lightweight DynaPort MT (106.6 × 58 × 11.5 mm, 55 g) consists of a tri-axial accelerometer (range: ±2 g, resolution: 1 mg), a tri-axial gyroscope (range: ±100 °/s, resolution: 0.0069 °/s), and a magnetometer (range: ±1000 μT, resolution: 0.10 μT). Data signals are stored on an in-built SD (secure digital) memory card at a sample frequency of 100 Hz. During raw data collection, the BFS communicates with a host computer via a Bluetooth connection. The supporting data acquisition software (DynaPort Manager V1.1.5; McRoberts B.V., The Hague, The Netherlands) was used to start and stop the BFS and to set event markers for the beginning and end of the 10 m steady-state walking trajectory. Marker placement during the test procedure was performed by the physical therapist using a Bluetooth remote controller. After the data collection, the DynaPort MT was connected to a computer with a USB cable and data recordings were uploaded to the central server of McRoberts B.V. for automatic data processing with established algorithms [33,58,59,60,61] through the standard online software module for gait analysis (DynaPort GaitTest; McRoberts B.V., The Hague, The Netherlands).

During the first test at T1, gait performance was additionally measured using the more advanced, multi-sensor based Mobility Lab (version 2) system (APDM Inc., Portland, OR, USA) as a reference standard [28]. Simultaneously to the DynaPort MT, participants wore three synchronized OPAL sensors attached with straps bilaterally on both feet and the fifth lumbar vertebra (Figure 1). The OPAL sensors (55 × 40.2 × 12.5 mm, <25 g) include two tri-axial accelerometers (range: ±16 g and ±200 g, resolution: 14 and 17.5 bits), gyroscope (range: ±2000 °/s, resolution: 12 bits) and magnetometer (range: ±8 Gauss, resolution 12 bits) and record at a sampling frequency of 128 Hz. The Mobility Lab system uses radio-frequency communication for wireless data transmission and synchronization of the multiple OPAL sensors through an access point connected to a host computer. The Mobility Lab (version 2) software (V2.0.0.201903301644; APDM Inc., Portland, OR, USA) was used to start and stop the OPAL sensors, to assign the OPAL sensors to the specific body site location (i.e., right/left foot, lumbar vertebra), to place event markers during data collection for the 10-m steady-state walking trajectory, and to automatically analyze the recorded data. Marker placement during the test procedure was performed by the physical therapist also using a Bluetooth remote controller.

Spatio-temporal gait parameters derived from the automatic output of the manufacturers provided (online) software modules included mean values of gait speed (m/s), step length (cm) and step time (s), cadence (steps/min), step time asymmetry (%), step time variability (s, standard deviation (SD) of step time) and walk ratio (cm/steps/min). Step time asymmetry and walk ratio were calculated as follows:

(1) $$\mathrm{Step}\;\mathrm{asymmetry}\;(\%)\;=\;100\;\times \;\frac{\left|{\mathrm{mean}\;\mathrm{step}\;\mathrm{time}}_{\mathrm{left}}\;-{\;\mathrm{mean}\;\mathrm{step}\;\mathrm{time}}_{\mathrm{right}}\right|}{{\mathrm{mean}\;\mathrm{step}\;\mathrm{time}}_{\mathrm{both}\;\mathrm{legs}}},$$

(2) $$\mathrm{Walk}\;\mathrm{ratio}\;(\mathrm{cm}/\mathrm{step}/\min)\;=\;\frac{\left|\mathrm{mean}\;\mathrm{step}\;\mathrm{length}\right|}{\mathrm{cadence}}.$$

### 2.5. Statistical Analysis

Descriptive data were presented as frequencies and percentages for categorical variables, and medians and ranges or means and SD for continuous variables as appropriate. The levels of agreement between the DynaPort MT and the Mobility Lab (concurrent validity) and the reproducibility between the repeated measurements at T1 (intra-session test-retest reliability) were assessed by calculating systematic between-method and test-retest differences (bias) with 95% confidence intervals (95% CI), 95% limits of agreement (LOA) as

(3) $$\mathrm{LOA}\;=\;{\mathrm{mean}}_{\mathrm{bias}}\;\pm \;1{.96\;\times \;\mathrm{SD}}_{\mathrm{bias}},$$

and ICC_2,1_ (two-way random single measure) with 95% CI for absolute agreement. ICC were interpreted as poor (<0.50), moderate (≥0.50 <0.75), good (≥0.75 <0.90), or excellent (>0.9) [62]. Bland–Altman plots were also constructed to visualize the levels of agreement between methods and the reproducibility between the repeated measurements [63]. In addition, percentage errors (PE) of the DynaPort MT compared to the Mobility Lab were calculated as

(4) $$\mathrm{PE}\;(\%)\;=\;100\;\times \;\frac{1{.96\;\times \;\mathrm{SD}}_{\mathrm{bias}}}{\frac{1}{2}\;\times \;\left({\mathrm{mean}}_{\mathrm{DynaPort}}{\;+\;\mathrm{mean}}_{\mathrm{MobilityLab}}\right)}.$$

PE were considered to be clinically acceptable if <30% [64]. Standard errors of measurement (SEM) were calculated by the square root of the mean square error terms from repeated-measures analyses of variance between test-retest measurements [65]. Minimal detectable changes (MDC) at the 95% CI were calculated as

(5) $${\mathrm{MDC}}_{95}=\;\mathrm{SEM}\;\times \;1.96\;\times \;\sqrt{2}.$$

SEM and MDC_95_ were also expressed as a percentage of the mean of all test-retest measurements:

(6) $$\mathrm{SEM}\%\;(\%{)}_{\;}=\;100\;\times \;\frac{\mathrm{SEM}\;}{{\mathrm{mean}}_{\mathrm{test}-\mathrm{retest}}},$$

(7) $${\mathrm{MDC}}_{95}\%\;(\%{)}_{\;}=\;100\;\times \;\frac{{\mathrm{MDC}}_{95}\;}{{\mathrm{mean}}_{\mathrm{test}-\mathrm{retest}}}.\;$$

An MDC_95_% of <30% was considered to be acceptable [66,67]. Sensitivity of change of the DynaPort MT was analyzed over the treatment period (T1 to T2) by calculating paired *t*-tests or Wilcoxon signed rank tests and standardized response means (SRM) as

(8) $$\mathrm{SRM}\;=\;\frac{{\mathrm{mean}}_{\mathrm{change}}}{{\mathrm{SD}}_{\mathrm{change}}}.$$

This analysis was performed for three subgroups of patients: those who experienced (1) an increase of ≥0.1-m/s (“improved patients”), (2) a decrease of ≥0.1-m/s (“worsened patients”), and (3) no change (smaller than ±0.1 m/s, “stable patients”) in the SPPB 4-m gait speed test. Subgroup classification was based on the minimal clinically important difference for change in usual gait speed [68,69,70]. SRM values were interpreted as trivial (<0.20), small (≥0.20 <0.50), moderate (≥0.5 <0.8), or large (≥0.8) [71]. Statistical significance was set at *p* < 0.05. Statistical analyses were performed using IBM SPSS Statistics for Windows, Version 24.0 (IBM Corp., Armonk, NY, USA).

## 3. Results

### 3.1. Participant Characteristics

The study sample included 58 acutely hospitalized older patients (83.7 ± 5.9 years), with about two-thirds being females (*n* = 39, 67.2%). Patients had multiple diagnoses (*n* = 9.9 ± 5.3) and took on average 11.1 ± 4.3 medications at admission. Forty-five (78.9%) patients reported one or more falls in the previous year. The MMSE score averaged 22.7 ± 4.7 points, with half of the patients (*n* = 29, 50.0%) having cognitive impairment (MMSE < 24 pt.). Almost half of the patients (*n* = 25, 43.1%) had clinically relevant depressive symptoms (GDS > 5 pt.). Fear of falling was low (FES-I = 16–19 pt.) in 20 (34.5%), moderate (FES-I = 20–27 pt.) in 19 (32.8%), and high (FES-I = 23−64 pt.) in 19 patients (32.8%). Physical capacity was low, with a mean SPPB score of 4.1 ± 1.6 points, a median DEMMI score of 52.2 ± 8.1 points, and a usual gait speed of 0.45 ± 0.18 m/s. Further participant characteristics are provided in Table 1.

### 3.2. Concurrent Valdity

**Figure 2:**
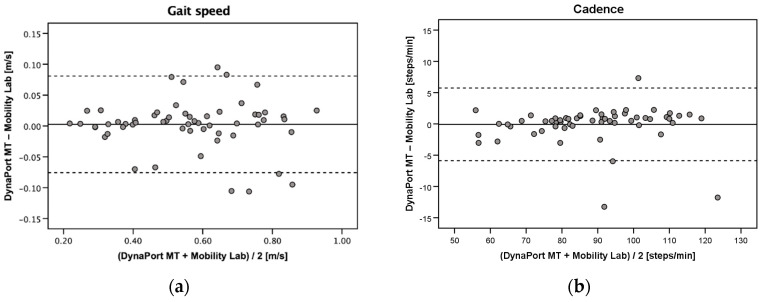

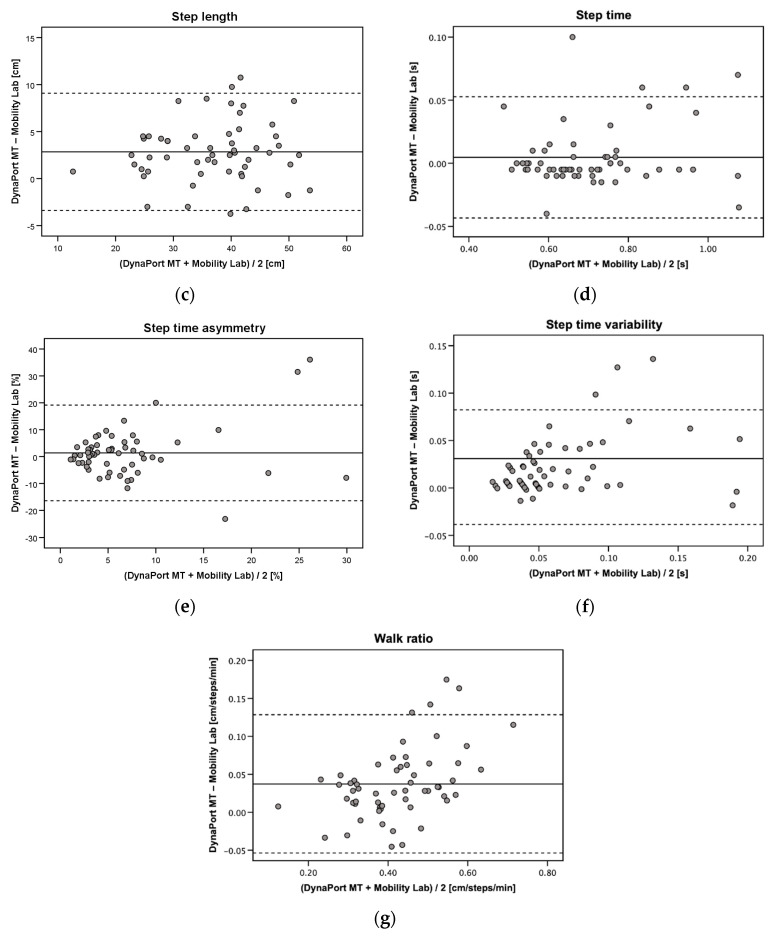
Bland–Altman plots for (**a**) gait speed, (**b**) cadence, (**c**) step length, (**d**) step time, (**e**) step time asymmetry, (**f**) step time variability, and (**g**) walk ratio measured with the DynaPort MT and the Mobility Lab (concurrent validity). Solid lines indicate mean between-method differences (bias) and dashed lines indicate upper and lower 95% limits of agreement (±1.96 SD of the bias).

Good to excellent absolute agreement between the DynaPort MT and the Mobility Lab was found for gait speed, cadence, step length, step time, and walk ratio (ICC_2,1_ = 0.87–0.99) (Table 2). Step time asymmetry (ICC_2,1_ = 0.29) and step time variability (ICC_2,1_ = 0.68) showed only poor to moderate agreement. PE were clinically acceptable for gait speed, cadence, step length, step time, and walk ratio (6.2–17.1%). Substantially higher and clinically not acceptable PE were observed for step time asymmetry (254.6%) and step time variability (95.2%) (Table 2). Bland–Altman plots for the agreement between the DynaPort MT and the Mobility Lab are provided in Figure 2a–g.

### 3.3. Test-Retest Reliability

**Figure 3:**
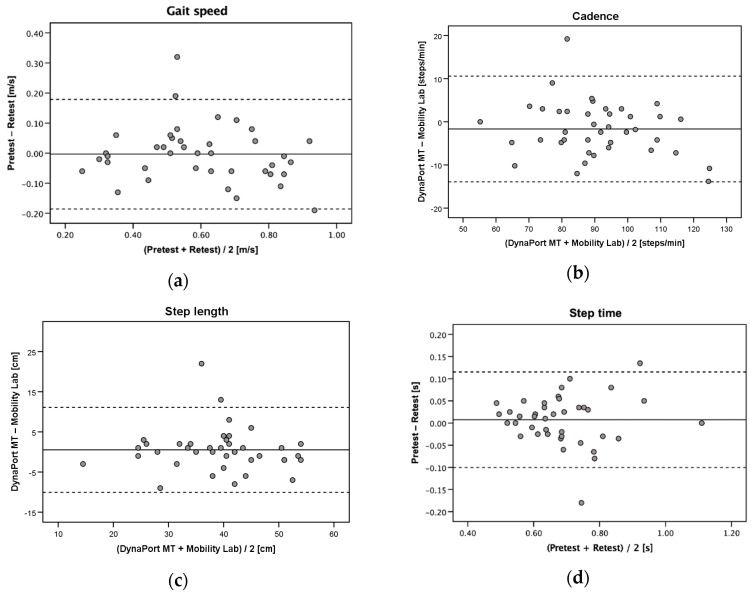

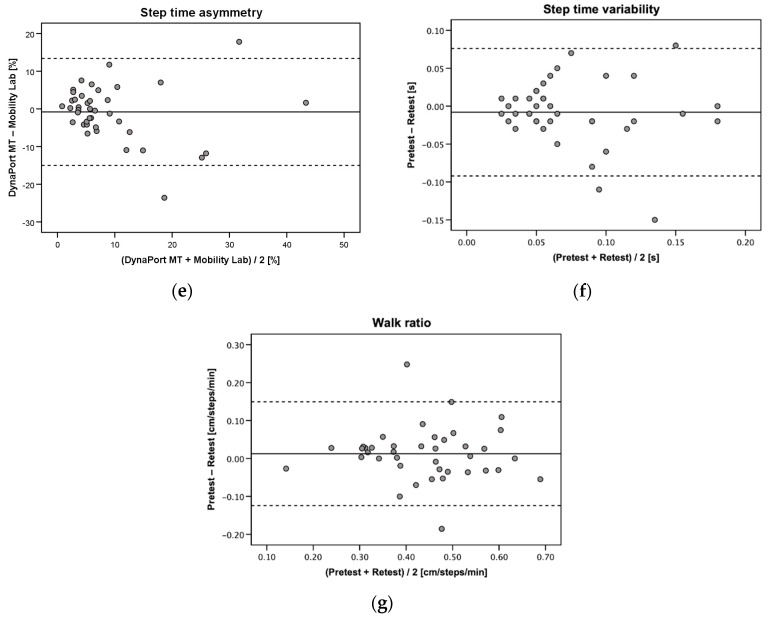
Bland–Altman plots for repeated measurements of (**a**) gait speed, (**b**) cadence, (**c**) step length, (**d**) step time, (**e**) step time asymmetry, (**f**) step time variability, and (**g**) walk ratio with the DynaPort MT (test-retest reliability). Solid lines indicate mean test-retest differences (bias) and dashed lines indicate upper and lower 95% limits of agreement (±1.96 SD of the bias).

Due to exhaustion which prevented a repeated measurement in 15 patients and invalid retest data in another 3 patients, test-retest reliability was assessed in a subsample of 40 patients. The DynaPort MT showed good to excellent test-retest reliability for gait speed, cadence, step length, step time, and walk ratio (ICC_2,1_ = 0.83–0.92) (Table 3). Only step time asymmetry (ICC_2,1_ = 0.72) and step time variability (ICC_2,1_ = 0.58) were below the threshold of good reliability (<0.75). Gait speed, cadence, step length, and walk ratio showed low SEM% (6.5–10.6%) and acceptable MDC_95_% (13.5–29.3%). Substantially higher SEM% values and not acceptable MDC_95_% values were observed for step time asymmetry (SEM% = 55.7%, MDC_95_% = 154.5%) and step time variability (SEM% = 42.3%, MDC_95_% = 117.2%). Bland–Altman plots for agreement between the repeated measurements with the DynaPort MT are provided in Figure 3a–g.

### 3.4. Sensitivity to Change

Sensitivity to change was assessed in 40 patients for whom data were available for gait analysis at T1 and T2. Eleven participants dropped out during the treatment period due to early discharge at own request (*n* = 7), transfer to another hospital (*n* = 3), or serious medical complications (*n* = 1); another seven patients were physically not able to complete (*n* = 5), refused the gait analysis at T2, despite repeated efforts of persuasion (*n* = 1), or had invalid data at T2 (*n* = 1).

A total of 22 (55%) patients were classified as improved, 12 (30%) as stable, and 6 (15%) as worsened according to their change in the 4 m gait speed test over the treatment period. Table 4 shows the results of the analysis of sensitivity to change by each patient subgroup.

For the improved subgroup, most gait parameters significantly improved (gait speed, cadence, step length, and walk ratio: *p* ≤ 0.001–0.011) or tended to be improved (step time: *p* = 0.061) from T1 to T2. Only step time asymmetry and variability were unchanged in this subgroup (*p* = 0.224–0.884). No significant within-group differences over the treatment period were found for the worsened (*p* = 0.093–0.893) and stable subgroup (*p* = 0.093–0.893). For the worsened subgroup, cadence and step time tended to be deteriorated at T2; however, missed the level of significance (*p* = 0.093–0.124).

SRM values were predominantly moderate to large for the improved subgroup, (gait speed, cadence, step length, and walk ratio: 0.62–0.99). The worsened subgroup also showed moderate to large SRM values for deteriorations in gait speed (−0.52), cadence (−0.85), and step time (−0.76). In both the improved and worsened subgroups, lowest SRM values were found for step time asymmetry and variability (−0.25–0.21). Compared to these two changed subgroups, the stable subgroup showed much smaller SRM values, ranging from trivial to small for all gait parameters (−0.04–0.40).

## 4. Discussion

The aim of this study was to evaluate the concurrent validity, test-retest reliability, and sensitivity to change of a single BFS system (DynaPort MT) for quantitative gait analysis in acute geriatric patients while walking with a rollator. To the best of our knowledge, the present study is the first to provide evidence on various psychometric properties of a single BFS for measuring spatio-temporal gait parameters under rollator-assisted walking conditions in such a vulnerable patient population.

### 4.1. Concurrent Validity

For evaluating the concurrent validity, the more advanced, rather high-cost multi-sensor based Mobility Lab was used as external reference standard for comparison, which has been already successfully used in geriatric patients for analyzing spatio-temporal gait parameters during rollator-assisted uphill, downhill, and level walking [72]. The ICC values indicate good to excellent absolute agreement of the DynaPort MT as a significantly lower-cost, single BFS system for measuring gait speed, cadence, step time, step length and walk ratio (ICC = 0.87–0.99).

In general, to our knowledge, no study has yet evaluated the psychometric properties of a single BFS system to assess gait parameters under rollator-assisted conditions, thus preventing a direct comparison of the present results with previous research. The only study evaluating the concurrent validity of a BFS system for gait analysis during rollator-assisted walking in geriatric patients reported similar high correlation coefficients (0.80–0.95) with a reference standard [42]. However, comparison with this study is difficult, as the BFS system consisted of a two IMU-based BFS systems, other spatio-temporal gait parameters were measured (stance, swing and stride time, stride length), another reference standard (GAITRite) was used, and correlation coefficients were given only for relative, but not for absolute level of agreement (Spearman’s rho). Other validation studies of single BFS systems (DynaPort MiniMod, FITMETER, Axivity AX3) attached to the lower back in healthy older adults reported similar high ICC values for absolute agreement with the GATIRite system for measuring gait speed, cadence, step time, and/or step length (ICC = 0.79–1.00) during normal walking without a mobility aid [37,73,74].

In contrast to the basic gait parameters of gait speed, cadence, step time, step length, and walk ratio, the levels of agreement for estimates of symmetry and variability between the DynaPort MT and the Mobility Lab were only poor to moderate (step time asymmetry: ICC = 0.29, step time variability, ICC = 0.68). This finding is similar to those reported in a recent systematic review on the validity and reliability of BFS systems for gait analysis in healthy adults, which identified spatio-temporal symmetry and variability parameters obtained from BFS systems to be less valid compared to spatio-temporal mean parameters [27]. Notably, there is, however, one study included showing excellent concurrent validity for the step time variability measured with a similar single BFS (DynaPort MiniMod) over separate walking trials (ICC = 0.88–0.94) [37]. As the validity of gait variability outcomes has been reported to improve with multiple walking trials [75], this test procedure may have improved these findings. In general, parameters addressing gait symmetry and variability based on individual footstep data have been discussed to be, by definition, more susceptible to random measurement errors from step to step as gait parameters on averaged footstep data which may mask such potential errors [27].

### 4.2. Test-Retest Reliability

Test-retest reliability of the DynaPort MT for measuring gait speed, cadence, step time, step length, and walk ratio was also good to excellent, as indicated by ICC values ranging from 0.83 to 0.92. The results on these gait parameters were quite similar or only slightly lower than those previously reported for the test-retest reliability of other single BFS systems placed on the lower back (e.g., FITMETER, DynaPort MiniMod, DynaPort Hybrid) in healthy young, middle-aged and older adults (ICC = 0.75–1.00) while walking without any walking aid [38,39,40,73,76].

The good reliability of these parameters was also demonstrated by their SEM% values, which were calculated to obtain the within-subject variability that typically occur due to random measurement error [65]. Although these SEM% values were higher compared to the previous studies in healthy (older) adults requiring no walking aid (1.9–4.7%) [38,40,76], they were predominantly lower than 10% (only the SEM% of walk ratio (10.1%) and gait speed (10.6%) were slightly higher), which is often considered to indicate an acceptable amount of a random measurement error [77,78]. Given the acute illnesses, comorbidity, and physical as well as cognitive impairments of our sample, which rather suggests a significantly higher variability in repeated physical capacity measurements compared to healthy adults, results of the test-retest reliability analyses are surprisingly good, demonstrating that various spatio-temporal gait parameters during rollator-assisted walking can be reliably obtained in acute geriatric patients by using the DynaPort MT.

In contrast to previous studies validating a single BFS system for gait analysis, we used the SEM values to provide MDC_95_ and MDC_95_% values for each gait parameter assessed with the DynaPort MT. These values offer clinicians and researchers the opportunity for determining whether a geriatric patient has experienced a real change in spatio-temporal gait parameters during rollator-assisted walking that exceed the measurement error, thus, making them highly relevant for evaluating treatment and intervention effects in clinical practice and research. We could identify only one study that provided similar information on MDC_95_ values for a multi-sensor BFS system (APDM Mobility Lab, version 1) in healthy younger adults without walking aids [79]. As expected, due to the considerably lower age and higher physical status, MDC_95_ values for cadence (2.7–3.7 steps/min), step length (0.07–0.11 m) and step time (0.03 s) were lower than those found in the current study for these gait parameters (cadence: 12.2 steps/min, step length: 10.6 cm, step time: 0.12 s).

Consistent with previous studies evaluating the test-retest reliability of single BFS-based gait analysis systems in healthy older adults [38,40,73], step time asymmetry (ICC = 0.72) and step time variability (ICC = 0.58) showed lower reproducibility than the basic gait parameters (gait speed, cadence, step length, step time, walk ratio). SEM% and MDC_95_% values were also substantially higher in these gait parameters (SEM% = 42.3–55.7%, MDC_95_% = 117.2–154.5%), with both clearly exceeding acceptable thresholds (SEM% < 10% [77,78], MDC_95_% < 30% [66,67]). Overall, the test-retest reliability of step time asymmetry and variability seem to be not sufficiently high for identifying within-subject differences and small intervention effects, thus questioning the value of these parameters captured by the DynaPort MT in clinical routine and research during rollator-assisted walking. A cautious use of the spatio-temporal gait symmetry and variability parameters has also been recommended in the above-mentioned systematic review, which found limited evidence not only on the validity but also on the reliability of BFS systems for measuring such parameters in healthy adult walking [27].

### 4.3. Sensitivity to Change

For use in clinical routine and research, it is essential an assessment instrument is able to detect changes over time or treatment and intervention effects. Independent of the specific walking condition analyzed in this study, to our knowledge, this study is the first that evaluated this psychometric property for a single BFS gait analysis system in acute geriatric patients. Sensitivity to change of the DynaPort MT was assessed in different subgroups of patients with improved, stable and worsened gait performance, as determined by the minimal clinically important difference for change in usual gait speed [68,69,70] assessed by established 4 m gait speed test of the SPPB [55]. This approach for assessing sensitivity to change in different subgroups was based on the assumption that the effect of the rehabilitation treatment would not be homogenous across the study sample of acutely hospitalized older patients for whom frequently a substantial decline in physical capacity and functional status have been reported [80,81,82,83,84]. Indeed, the study sample showed heterogeneous treatment effects on usual gait speed, with 55% of them improving, 30% remaining stable, and 15% worsening. The significant improvements for most gait parameters (gait speed, cadence, step length, walk ratio) with moderate to large SRM values (0.62–0.99) in the improved subgroup demonstrated the high potential of the DynaPort MT to detect positive changes during rollator-assisted walking in acute geriatric patients. Slightly lower, but also moderate to large SRM values (−0.85 to −0.52), were observed for deteriorations in gait speed, cadence, and step time among the worsened subgroup; however, these negative changes missed the level of significance, which might have been related to the small number of patients in this subgroup. Overall, these results suggest that the DynaPort MT was more sensitive to improvements than to deteriorations in gait performance.

The non-significant changes and low SRM values for step time asymmetry and variability in the improved and worsened subgroups might be related to the low reliability of the DynaPort MT for measuring these gait parameters, which illustrates once more the limited suitability of these parameters for detect real changes.

### 4.4. Limitations

This study has some limitations. First, our results are restricted to walking with a standard rollator (four-wheeled walker) in geriatric patients, limiting their generalizability to other assisted walking conditions and patient populations. Future studies should evaluate the psychometric properties of a single BFS for gait analysis when using other walking aids (e.g., walking frames, canes) or during rollator-assisted walking in other persons who need a rollator for walking. Second, the more-advanced, multi-sensor based Mobility Lab was used as an external reference standard, while previous studies on the concurrent validity of BFS systems for gait analysis most frequently used the GAITRite system for comparison [27]. However, the Mobility Lab has demonstrated good to excellent concurrent validity with the GAITRite system [28,31]. Finally, test-retest reliability was assessed from within-session measurements, and may not be represent variation in repeated rollator-assisted gait performance over longer periods of time.

## 5. Conclusions

The results of this study showed that the DynaPort MT as a single BFS system has good to excellent concurrent validity compared with the multi-sensor based APDM Mobility as well as good to excellent test-retest reliability for the measurement of gait speed, cadence, step length, step time, and walk ratio during rollator-assisted walking in acutely hospitalized older patients. Results also demonstrated that for most of these gait parameters the DynaPort MT is sensitive to detect changes in gait performance over the course of early geriatric rehabilitation treatment, whereby it appears to be more sensitive to improvements in gait performance than to deteriorations. Parameters of gait variability and asymmetry need to be viewed with caution as they consistently showed lower psychometric properties, with only poor to moderate concurrent validity with the Mobility Lab, poor to moderate test-retest reliability, and trivial to small sensitivity to change. Overall, the DynaPort MT as a highly portable, affordable and easy-to-use instrument can provide clinicians and researchers with a set of valid, reliable and sensitive spatio-temporal gait parameters for quantitative gait analysis in acute geriatric patients who can only walk with a rollator.

## Figures and Tables

**Table 1:** Participant characteristics.

Variables	*n* = 58
Age [years]	83.7 ± 5.9
Sex [female]	39 (67.2)
Diagnoses [*n*]	9.9 ± 5.3
Medications [*n*]	11.1 ± 4.3
Fall in the previous year ^1^ [*n*]	45 (78.9)
Mini-Mental State Examination [pt.]	22.7 ± 4.7
Geriatric Depression Scale [pt.]	5.3 ± 3.3
Falls Efficacy Scale-International [pt.]	10 [7–26]
EuroQol-5 Dimensions [pt.]	0.68 ± 0.24
Barthel Index [pt.]	72.1 ± 20.1
Short Physical Performance Battery [pt.]	4.3 ± 1.7
Gait speed [m/s]	0.45 ± 0.18
De Morton Mobility Index [pt.]	52.2 ± 8.1
Handgrip strength [kg]	16.9 ± 7.7

Data are given as mean ± standard deviation, *n* (%), or median [range]. ^1^ based on *n* = 57 due to missing information for one participant.

**Table 2:** Concurrent validity of the DynaPort MT with the Mobility Lab (*n* = 58).

Parameter	DynaPort MT (Mean ± SD)	Mobility Lab (Mean ± SD)	Bias (95% CI)	95% LOA	PE	ICC_2,1_ (95% CI)
Gait speed [m/s]	0.56 ± 0.18	0.56 ± 0.18	0.00 (–0.01 to 0.01)	–0.08 to 0.08	13.9	0.98 (0.96 to 0.99)
Cadence [steps/min]	88.4 ± 16.5	88.5 ± 16.4	–0.1 (–0.9 to 0.7)	–5.9 to 5.7	6.6	0.98 (0.97 to 0.99)
Step length [cm]	38.0 ± 9.1	35.1 ± 8.9	2.8 (2.0 to 3.7)	–3.4 to 9.1	17.1	0.89 (0.55 to 0.96)
Step time [s]	0.71 ± 0.15	0.71 ± 0.14	0.00 (–0.01 to 0.00)	–0.09 to 0.10	6.8	0.99 (0.98 to 0.99)
Step time asymmetry [%]	7.67 ± 8.56	6.30 ± 6.60	1.37 (–1.02 to 3.75)	–16.41 to 19.15	254.6	0.29 (0.04 to 0.51)
Step time variability [s]	0.07 ± 0.05	0.05 ± 0.04	0.02 (0.01 to 0.03)	–0.04 to 0.08	95.2	0.68 (0.31 to 0.84)
Walk ratio [cm/steps/min]	0.44 ± 0.12	0.40 ± 0.10	0.04 (–0.05 to 0.03)	–0.05 to 0.13	21.5	0.87 (0.56 to 0.95)

SD, standard deviation; CI, confidence interval; LOA, limits of agreement; PE, percentage error, ICC, intraclass correlation coefficient.

**Table 3:** Intra-session test-retest reliability of the DynaPort MT (*n* = 40).

Parameter	Pretest (Mean ± SD)	Retest (Mean ± SD)	Bias (95% CI)	95% LOA	ICC_2,1_ (95% CI)	SEM (SEM%)	MDC_95_ (MDC_95_%)
Gait speed [m/s]	0.60 ± 0.18	0.60 ± 0.20	0.00 (–0.03 to 0.03)	–0.19 to 0.18	0.89 (0.79 to 0.94)	0.06 (10.6)	0.18 (29.3)
Cadence [steps/min]	90.1 ± 15.3	91.7 ± 16.7	–1.7 (–3.7 to 0.3)	–13.9 to 10.6	0.92 (0.86 to 0.96)	4.4 (4.9)	12.2 (13.5)
Step length [cm]	39.4 ± 9.4	38.9 ± 9.7	0.5 (–1.2 to 2.3)	–10.1 to 11.1	0.84 (0.72 to 0.91)	3.8 (9.8)	10.6 (27.0)
Step time [s]	0.69 ± 0.13	0.68 ± 0.13	0.01 (–0.01 to 0.03)	–0.12 to 0.10	0.91 (0.84 to 0.95)	0.04 (6.5)	0.12 (18.0)
Step time asymmetry [%]	8.8 ± 9.2	9.6 ± 9.9	–0.8 (–3.1 to 1.5)	–15.0 to 13.4	0.72 (0.53 to 0.84)	5.1 (55.7)	14.2 (154.5)
Step time variability [s]	0.07 ± 0.04	0.08 ± 0.06	–0.01 (–0.02 to 0.01)	–0.09 to 0.08	0.58 (0.33 to 0.75)	0.03 (42.3)	0.09 (117.2)
Walk ratio [cm/steps/min]	0.45 ± 0.12	0.44 ± 0.12	0.01 (–0.01 to 0.03)	–0.12 to 0.15	0.83 (0.71 to 0.91)	0.04 (10.1)	0.12 (28.1)

SD, standard deviation; CI, confidence interval; LOA, limits of agreement; ICC, intraclass correlation coefficient; SEM, standard error of measurement; MDC, minimal detectable change.

**Table 4:** Sensitivity to change of the DynaPort MT in improved (*n* = 22), stable (*n* = 12) and worsened patients (*n* = 6).

Parameter	T1 (Mean ± SD)	T2 (Mean ± SD)	*p*-Value ^1^	SRM
Gait speed [m/s]				
Improved	0.54 ± 0.17	0.66 ± 0.18	<0.001	0.99
Stable	0.54 ± 0.19	0.57 ± 0.22	0.389	0.26
Worsened	0.65 ± 0.21	0.58 ± 0.20	0.260	−0.52
Cadence [steps/min]				
Improved	91.1 ± 12.3	96.6 ± 8.1	0.011	0.60
Stable	84.7 ± 18.0	84.4 ± 16.2	0.923	−0.03
Worsened	90.0 ± 21.9	84.0 ± 20.9	0.093	−0.85
Step length [cm]				
Improved	35.0 ± 9.9	41.5 ± 11.4	<0.001	0.97
Stable	37.4 ± 9.2	39.5 ± 9.8	0.192	0.40
Worsened	43.2 ± 4.8	41.0 ± 6.8	0.387	−0.39
Step time [s]				
Improved	0.68 ± 0.11	0.64 ± 0.05	0.061	−0.42
Stable	0.74 ± 0.16	0.75 ± 0.17	0.852	0.06
Worsened	0.71 ± 0.20	0.75 ± 0.19	0.124	0.76
Step time asymmetry [%]				
Improved	8.4 ± 9.8	7.3 ± 7.3	0.224	−0.09
Stable	6.9 ± 6.7	6.6 ± 5.2	>0.999	−0.04
Worsened	11.7 ± 14.5	12.0 ± 12.6	0.893	0.02
Step time variability [s]				
Improved	0.09 ± 0.08	0.06 ± 0.05	0.884	−0.25
Stable	0.07 ± 0.05	0.09 ± 0.10	0.822	0.30
Worsened	0.07 ± 0.05	0.08 ± 0.03	0.854	0.21
Walk ratio [cm/min/steps]				
Improved	0.39 ± 0.12	0.43 ± 0.13	0.008	0.62
Stable	0.45 ± 0.11	0.48 ± 0.11	0.219	0.38
Worsened	0.50 ± 0.14	0.51 ± 0.14	0.748	0.14

^1^*p*-values are given for paired *t*-tests (gait speed, cadence, step length, step time, and walk ratio) or Wilcoxon signed rank tests (step time asymmetry and variability). T1, hospital admission; T2; hospital discharge; SRM, standard response mean; SD, standard deviation.
